# Successful resolution of extensive tuberculosis verrucosa cutis with local antituberculous therapy regime

**DOI:** 10.1016/j.jdcr.2026.03.007

**Published:** 2026-03-13

**Authors:** Juan Luis Galán Flores, Raúl Vicente Cabezas Echegoyen, Alexandra Maza, Francisca Flores de Galán, Silvia Anett Mejía Rodríguez

**Affiliations:** a Dermatology Resident (R4), Dermatology Center, National Zacamil Hospital “Dr. Juan José Fernández”, San Salvador, El Salvador; b Dermatology Resident (R3), Dermatology Center, National Zacamil Hospital “Dr. Juan José Fernández”, San Salvador, El Salvador; c Dermatopathology, Private Practice, San Salvador, El Salvador; d Dermatology, Internal Medicine, Dermaplast Clinic, San Salvador, El Salvador; e Pediatric Dermatology, Private Practice, San Salvador, El Salvador

**Keywords:** antituberculous agents, bacterial, cutaneous, diabetes mellitus, granuloma, mycobacterium tuberculosis, skin diseases, tuberculosis, type 2

## Case description

**Fig 1:**
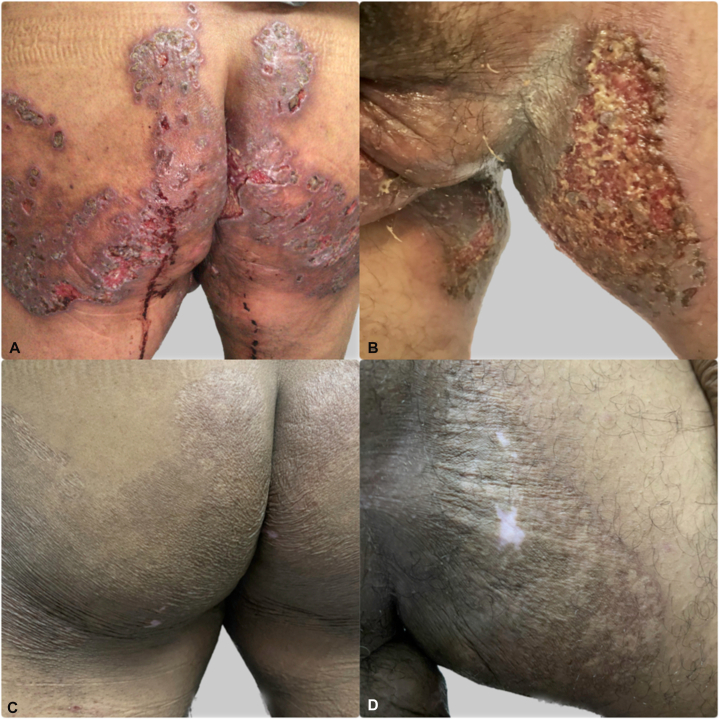
Clinical views: **(A** and **B)**, posterior and anterior views respectively, showing the initial clinical appearance before treatment. November 2020. **C** and **D,** The posterior and anterior views show clinical resolution after treatment completion. October 2021.

**Fig 2:**
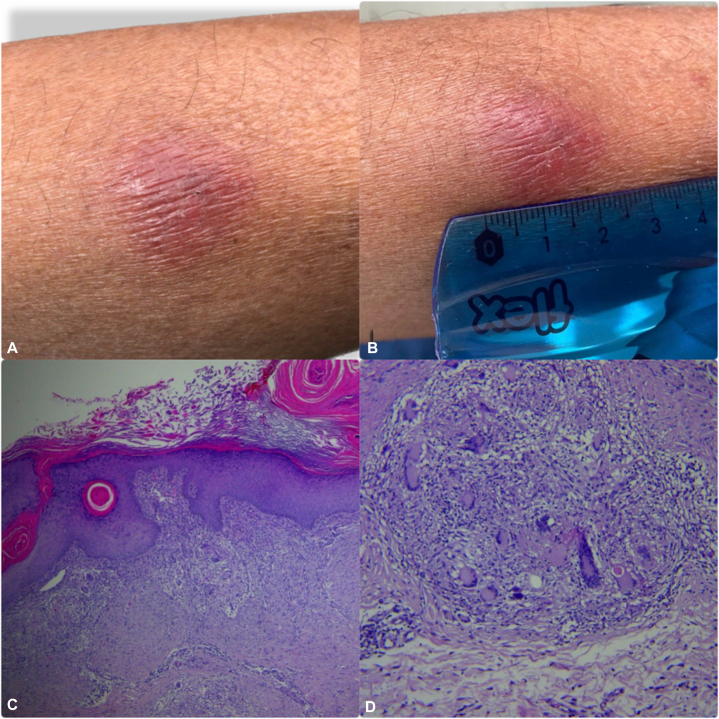
Tuberculin skin test **(A** and **B)**. Skin biopsy, H&E stain **(C** and **D)**. **A,** Skin induration and erythema after applying TST. **B,** Positive result of TST, measuring approximately 20 mm. **C,** H&E-4×, verrucous hyperplasia, diffuse dermal granulomatous inflammation. **D,** H&E-20×, presence of multinucleated Langhans-type giant cells. *H&E*, Hematoxylin and eosin; *TST*, tuberculin skin test.

A 55-year-old man presented with an 18-month slowly progressive eruption that began on the right buttock and extended to the medial thighs, contralateral buttock, groin, perineum, and scrotum. Comorbidities included type 2 diabetes mellitus and chronic alcohol use. Examination showed bilateral, symmetric confluent ulcerated plaques (4-12 cm) with purulent-sanguineous exudate, meliceric and hemorrhagic crusts, and fibrinous debris (Fig 1, *A* and *B*). A tuberculin skin test was strongly positive (20 mm; Fig 2, *A* and *B*), and the chest radiograph was normal. Skin biopsy revealed pseudoepitheliomatous epidermal hyperplasia and chronic granulomatous inflammation with Langhans-type giant cells; acid-fast bacilli were not seen on Ziehl-Neelsen stain (Fig 2, *C* and *D*). HIV and syphilis serologies were negative, and he had received Bacillus Calmette–Guérin vaccination in childhood. Mycobacterial culture and polymerase chain reaction from skin were unavailable. He received a 6-month antituberculosis regimen, consisting of isoniazid, rifampicin, pyrazinamide, and ethambutol for 2 months, followed by isoniazid and rifampicin for 4 months, according to the El Salvador tuberculosis (TB) program. Complete clinical resolution was noted at 6-month follow-up (Fig 1 *C* and *D*).

**Question: What is the most likely route of infection in this case of cutaneous tuberculosis (CTB)?**

A. Inoculation tuberculosis.
B. Contiguous spread tuberculosis.
C. Hematogenous spread tuberculosis.
D. Autoinoculation tuberculosis.
E. Lymphatic spread tuberculosis.

## Discussion

Correct answer: **A**. Inoculation tuberculosis.

Cutaneous tuberculosis (CTB) is a type of extrapulmonary tuberculosis (EPTB) and represents approximately 1.5% to 3% of all TB cases. Tuberculosis verrucosa cutis (TBVC) accounts for 3% to 19% of CTB cases.^1^^,^^2^ Regarding El Salvador, data are only available for EPTB; however, no official national data are available on CTB. TBVC is acquired by primary inoculation of the skin or mucosa with Mycobacterium tuberculosis in a previously sensitized, immunocompetent host and is typically paucibacillary.^1^, ^2^, ^3^ In a 50-case series, TBVC predominated in younger patients, most often with acral or buttock lesions, and type 2 diabetes mellitus was the most frequent comorbidity.^3^ Our patient’s profile partially fits these findings, but his older age and extensive perineogenital involvement broaden the classical spectrum and highlight the need to recognize TBVC in atypical distributions. Type 2 diabetes mellitus and chronic alcohol use are risk factors for pulmonary TB, through impaired macrophage function, cell-mediated immunity, and nutritional dysregulation; these conditions may likely increase the susceptibility for developing CTB. More severe or disseminated multibacillary forms of CTB are usually observed in profoundly immunocompromised hosts.

Diagnosis of CTB is challenging because it can mimic infectious skin diseases, such as deep mycoses, leishmaniasis, and actinomycosis, as well as noninfectious entities, such as hypertrophic lichen planus or squamous cell carcinoma.^1^^,^^2^ Mycobacterial culture remains the gold standard for species identification and drug susceptibility testing, but it is slow and often negative in paucibacillary TBVC; culture and polymerase chain reaction from skin were unavailable in this case.^4^ Whenever feasible, a multimodal evaluation is advisable for patients with CTB. This includes clinicopathologic correlation; imaging to screen for extracutaneous involvement (eg, chest radiography, with further imaging guided by symptoms) and to define the extent of cutaneous disease when deep involvement; assessment of host risk factors (eg, HIV testing, nutritional status); and microbiologic/immunologic testing (mycobacterial culture and/or polymerase chain reaction when available, plus tuberculin skin test or interferon-gamma release).^1^^,^^2^^,^^4^ In resource-limited settings, or when ancillary tests are inconclusive, a carefully monitored therapeutic trial of antituberculous therapy may be justified. Early clinical improvement supports CTB. A lack of response should prompt reassessment for alternative diagnoses, particularly infectious mimickers not covered by standard antituberculous therapy.^4^^,^^5^ Our patient’s rapid and sustained response to a 6-month regimen with antituberculosis drugs illustrates how empiric therapy, when guided by strong clinicopathologic evidence and thoughtful exclusion of mimickers, can serve as both a diagnostic and therapeutic approach for TBVC in resource-limited settings.

## Conflicts of interest

None disclosed.
